# Prevalence and Severity of Food Allergies Among US Adults

**DOI:** 10.1001/jamanetworkopen.2018.5630

**Published:** 2019-01-04

**Authors:** Ruchi S. Gupta, Christopher M. Warren, Bridget M. Smith, Jialing Jiang, Jesse A. Blumenstock, Matthew M. Davis, Robert P. Schleimer, Kari C. Nadeau

**Affiliations:** 1 Institute for Public Health and Medicine, Northwestern University Feinberg School of Medicine, Chicago, Illinois; 2 Department of Pediatrics, Northwestern University Feinberg School of Medicine, Chicago, Illinois; 3 Mary Ann & J. Milburn Smith Child Health Research, Outreach, and Advocacy Center, Ann & Robert H. Lurie Children’s Hospital, Chicago, Illinois; 4 Department of Medicine, Northwestern University Feinberg School of Medicine, Chicago, Illinois; 5 Department of Preventive Medicine, University of Southern California Keck School of Medicine, Los Angeles; 6 Center for Innovation for Complex Chronic Healthcare, Edward J. Hines Jr Veterans Affairs Hospital, Hines, Illinois; 7 Department of Medical Social Sciences, Northwestern University Feinberg School of Medicine, Chicago, Illinois; 8 Department of Preventive Medicine, Northwestern University Feinberg School of Medicine, Chicago, Illinois; 9 Sean N. Parker Center for Allergy and Asthma Research, Stanford University School of Medicine, Stanford, California

## Abstract

**Importance:**

Food allergy is a costly, potentially life-threatening condition. Although studies have examined the prevalence of childhood food allergy, little is known about prevalence, severity, or health care utilization related to food allergies among US adults.

**Objective:**

To provide nationally representative estimates of the distribution, severity, and factors associated with adult food allergies.

**Design, Setting, and Participants:**

In this cross-sectional survey study of US adults, surveys were administered via the internet and telephone from October 9, 2015, to September 18, 2016. Participants were first recruited from NORC at the University of Chicago’s probability-based AmeriSpeak panel, and additional participants were recruited from the non–probability-based Survey Sampling International (SSI) panel.

**Exposures:**

Demographic and allergic participant characteristics.

**Main Outcomes and Measures:**

Self-reported food allergies were the main outcome and were considered convincing if reported symptoms to specific allergens were consistent with IgE-mediated reactions. Diagnosis history to specific allergens and food allergy–related health care use were also primary outcomes. Estimates were based on this nationally representative sample using small-area estimation and iterative proportional fitting methods. To increase precision, AmeriSpeak data were augmented by calibration-weighted, non–probability-based responses from SSI.

**Results:**

Surveys were completed by 40 443 adults (mean [SD] age, 46.6 [20.2] years), with a survey completion rate of 51.2% observed among AmeriSpeak panelists (n = 7210) and 5.5% among SSI panelists (n = 33 233). Estimated convincing food allergy prevalence among US adults was 10.8% (95% CI, 10.4%-11.1%), although 19.0% (95% CI, 18.5%-19.5%) of adults self-reported a food allergy. The most common allergies were shellfish (2.9%; 95% CI, 2.7%-3.1%), milk (1.9%; 95% CI, 1.8%-2.1%), peanut (1.8%; 95% CI, 1.7%-1.9%), tree nut (1.2%; 95% CI, 1.1%-1.3%), and fin fish (0.9%; 95% CI, 0.8%-1.0%). Among food-allergic adults, 51.1% (95% CI, 49.3%-52.9%) experienced a severe food allergy reaction, 45.3% (95% CI, 43.6%-47.1%) were allergic to multiple foods, and 48.0% (95% CI, 46.2%-49.7%) developed food allergies as an adult. Regarding health care utilization, 24.0% (95% CI, 22.6%-25.4%) reported a current epinephrine prescription, and 38.3% (95% CI, 36.7%-40.0%) reported at least 1 food allergy–related lifetime emergency department visit.

**Conclusions and Relevance:**

These data suggest that at least 10.8% (>26 million) of US adults are food allergic, whereas nearly 19% of adults believe that they have a food allergy. Consequently, these findings suggest that it is crucial that adults with suspected food allergy receive appropriate confirmatory testing and counseling to ensure food is not unnecessarily avoided and quality of life is not unduly impaired.

## Key Points

**Question:**

What are the prevalence and severity of food allergy in US adults?

**Findings:**

In a population-based survey study of 40 443 US adults, an estimated 10.8% were food allergic at the time of the survey, whereas nearly 19% of adults believed that they were food allergic. Nearly half of food-allergic adults had at least 1 adult-onset food allergy, and 38% reported at least 1 food allergy–related emergency department visit in their lifetime.

**Meaning:**

The findings suggest that food allergies are common and severe among US adults, often starting in adulthood.

## Teaser

This survey study provides nationally representative estimates of the distribution, severity, and factors associated with adult food allergy in the United States.

## Introduction

Food allergy is a costly,^1^ potentially life-threatening^2^ health condition that can adversely affect patients’ well-being.^3,4^ Although population-based studies^5,6^ have examined the prevalence of food allergy among children, less is known about the population-level burden of food allergy among adults in the United States. The few population-based studies^7,8^ to date that examined adult food allergy have focused on a limited number of specific allergens (eg, peanut) or allergen groups (eg, tree nut, seafood) or have been secondary analyses of federal health surveys, which were not designed to comprehensively characterize food allergy prevalence and severity among US adults. For example, neither the Centers for Disease Control and Prevention’s National Health and Nutrition Examination Survey^9^ nor the US Food and Drug Administration’s (FDA’s) Food Safety Survey^10^ collects information about specific allergic reaction symptoms critical for differential diagnosis of food allergy (eg, food intolerances, oral allergy syndrome). Nevertheless, food allergy prevalence estimates from these recent national surveys exceed 9% of US adults, suggesting that food allergy may affect more US adults than previously acknowledged.

Although some children with food allergy develop natural tolerance, others retain their food allergy as they enter adulthood.^11,12^ Adults can also develop new food allergies,^13^ and evidence suggests that certain food allergies (eg, shellfish and fin fish) may be more likely than others to develop during adulthood.^8,13^ Moreover, studies^14,15,16^ suggest that rates of food allergy–related emergency department (ED) visits may be increasing among children and young adults.

Much remains to be learned about the population-level consequences of adult food allergy in the United States, including the relative frequency and timing of adult- vs childhood-onset food allergy, allergen type, severity, and key sociodemographic and clinical factors of each of these food allergy characteristics. This study aimed to provide comprehensive, nationally representative estimates of the distribution, severity, and factors associated with adult food allergy in the United States.

## Methods

Surveys were administered by NORC at the University of Chicago from October 9, 2015, to September 18, 2016, to a sample of US households through a dual-sampling approach using NORC’s nationally representative AmeriSpeak panel and the Survey Sampling International (SSI) non–probability-based sample (eMethods in the Supplement). Written informed consent was obtained from all participants during enrollment into the AmeriSpeak panel and SSI web samples. Identical surveys were administered to both samples. All data were deidentified. The NORC Institutional Review Board and Northwestern University Institutional Review Board approved all study activities. The study followed the American Association for Public Opinion Research (AAPOR) reporting guideline.

### Survey Development and Design

The surveys extended our national child food allergy survey, administered in 2009 to 2010, which was developed by pediatricians, allergists, health services researchers, and survey methodologists. Expert panel review and key informant cognitive interviews (N = 40) were conducted on the original survey using the approach described previously.^17^ Although core constructs from the 2009-2010 survey were retained, additional questions were added to the present instrument to assess emerging research issues that related to the cause and management of adult food allergy. The revised instrument was pretested on 345 interviewees to ensure clarity, relevance, validity, and reliable functioning of all questions and response options. Interviewee data and feedback were reviewed and incorporated into the final 2015-2016 surveys, which were administered via the internet or telephone. All write-in responses were hand coded and reviewed by an expert panel to ensure accuracy of final data. Participants who did not answer the initial question about whether they have ever had a food allergy were considered to have provided incomplete responses and were not included in any analyses.

### Outcome Measures

**Figure 1.**
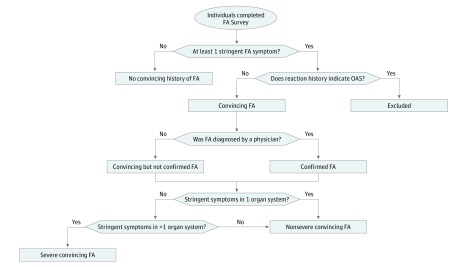
Convincing, Physician-Diagnosed, and Severe Food Allergy (FA) Categorization Flow Diagram Stringent symptoms by organ system include skin or oral mucosa (hives, swelling [except lip or tongue], lip or tongue swelling, difficulty swallowing, throat tightening), respiratory tract (chest tightening, trouble breathing, wheezing), gastrointestinal tract (vomiting), and cardiovascular (chest pain, rapid heartbeat, fainting, low blood pressure). Gastrointestinal symptoms commonly associated with intolerance (eg, diarrhea, cramps) were not considered to be stringent symptoms. The following allergies were considered for exclusion as probable oral allergy syndrome (OAS) based on symptom report: fruit, vegetable, peanut, tree nut, wheat, soy, barley, rice, seed, spice, shellfish, and fin fish.

The primary outcome measures for the study were the prevalence and severity of overall and food-specific convincing adult food allergy. Food allergies were considered to be convincing if the most severe reaction reported to that food included at least 1 symptom on the stringent symptom list developed by our expert panel (eFigure in the Supplement). Reported allergies with reaction symptoms characteristic of oral allergy syndrome or food intolerances were excluded and not considered to be convincing according to the food allergy categorization flowchart summarized in Figure 1, even if such allergies were reported as diagnosed by a physician. Only convincing food allergies for which a physician’s diagnosis was reported were considered to be physician diagnosed for the purposes of our study. For each convincing allergy, a severe reaction history was indicated by reporting 1 or more stringent symptoms across 2 or more of the following organ systems: skin or oral mucosa, gastrointestinal tract, cardiovascular, and respiratory tract.

If multiple food allergies were reported, each reported food allergy was evaluated separately using the food allergy categorization flowchart. For example, if a respondent reported a nut allergy with a reaction history limited to oral symptoms indicative of oral allergy syndrome as well as a shellfish allergy with a reaction history that included throat tightening, vomiting, and hives, the respondent would be considered to have only a single, severe shellfish allergy and the nut allergy would be excluded. Lifetime physician-diagnosed atopic comorbidities were also assessed using the question, “Have you ever been diagnosed by a doctor with any of the following chronic conditions? Please select all that apply.” Response options included asthma, eczema/atopic dermatitis, hay fever/allergic rhinitis/seasonal allergies, insect sting allergy, latex allergy, medication allergy, and urticaria/chronic hives.

### Study Participants and Survey Weighting

Eligible study participants included adults (≥18 years of age) able to complete surveys in English or Spanish who were residing in a US household. As in the 2009-2010 survey, this study relied on a nationally representative household panel to support population-level inference.^5^ Study participants were first recruited from NORC at the University of Chicago’s probability-based AmeriSpeak panel, where a survey completion rate of 51.2% was observed (7218 responses from 14 095 invitees). The weighted cumulative AAPOR response rate for the AmeriSpeak sample was 8.8%. This rate is a function of the 18.3% rate of originally sampled households successfully recruited into the AmeriSpeak panel when it was established, the 93.8% rate of successfully recruited households who were also successfully retained into the panel so that they were potentially eligible for participation in the present study, and the aforementioned 51.2% completion rate among successfully recruited and retained AmeriSpeak panelists who were approached for this particular study. Each AmeriSpeak respondent was assigned a base, nonresponse-adjusted sampling weight, which was then ranked to external population totals associated with age, sex, educational level, race/ethnicity, housing tenure, telephone status, and census division using iterative proportional fitting to improve external validity. To increase precision of estimates when data were scarce, such as for the prevalence of rare allergies within specific age groups, and ensure sufficient sample size among key subpopulations, prevalence estimates calculated from population-weighted AmeriSpeak responses were augmented by calibration-weighted, non–probability-based responses obtained through the SSI Dynamix platform.^18^ SSI is a leading survey research organization with a diverse and large web-based panel of potential participants, who were sampled for the present study using methods designed to minimize self-selection bias. State-of-the-art small-area estimation methods were used, which leverage similarity and borrow strength across all available information in both samples to minimize the bias and variance of resulting estimates to a greater degree than independent analysis of either sample permitted.^19^ These methods are frequently used by census bureaus and national survey research organizations because of their efficiency and effectiveness.^20,21^ The final, combined sample weight was derived by applying an optimal composition factor that minimizes the mean square error associated with food allergy prevalence estimates. In total, surveys were completed by 40 443 US adults, each of whom received $5 on survey completion.

### Statistical Analysis

Complex survey weighted proportions and 95% CIs were calculated to estimate prevalence using the svy: tabulate command using the “ci” and “per” options in Stata statistical software, version 14 (StataCorp).^22^ Relative proportions of demographic characteristics were compared using weighted Pearson χ^2^ statistics, which were corrected for the complex survey design with the second-order correction of Rao and Scott^23^ and converted into *F* statistics. Covariate-adjusted complex survey weighted logistic regression models compared relative prevalence and other assessed food allergy outcomes by participant characteristics. Two-sided hypothesis tests were used, with 2-sided *P* < .05 considered to be statistically significant.

## Results

### Demographics, Food Allergy Prevalence, and Childhood vs Adult-Onset Allergies

Surveys were completed by 40 443 adults (7210 from the AmeriSpeak panel and 33 233 from the SSI panel; mean [SD] age, 46.6 [20.2] years). As anticipated, the observed completion rate was higher among the probability-based AmeriSpeak panel (51.2% of invited adults) compared with the non–probability-based SSI panel (5.5% of invited adults). The weighted distributions of respondents by age, sex, and race/ethnicity (eTable 1 in the Supplement) were consistent with 2016 estimates from the US Census Bureau’s Current Population Survey.^24^

Overall, 10.8% (95% CI, 10.4%-11.1%) of US adults were estimated to have 1 or more current convincing food allergies. However, an estimated 19.0% (95% CI, 18.5%-19.5%) of US adults reported at least 1 convincing or nonconvincing FA. (Table 1). Among all adults with convincing food allergy, 48.0% (95% CI, 46.2%-49.7%) reported developing at least 1 of their convincing food allergies as an adult, whereas 26.9% (95% CI, 25.3%-28.6%) developed convincing food allergy only during adulthood and 52.0% (95% CI, 50.3%-53.8%) developed convincing food allergy only before 18 years of age.

**Table 1.** Estimated Current FA Prevalence Rates Among US Adults

Variable	Prevalence of Current FA, % (95% CI)	*P* Value	Prevalence of Adult-Onset Current FA, % (95% CI)	*P* Value
Overall	10.8 (10.4-11.1)	NA	5.2 (4.9-5.4)	NA
Race/ethnicity				
Asian, non-Hispanic	11.4 (9.8-13.3)	<.001	4.8 (3.8-6.1)	<.001
Black, non-Hispanic	11.2 (10.2-12.3)		5.1 (4.4-5.9)	
White, non-Hispanic	10.1 (9.7-10.6)		5.2 (4.9-5.5)	
Hispanic	11.6 (10.5-12.8)		4.6 (3.9-5.4)	
Multiple or other	15.9 (13.6-18.6)		7.2 (5.8-9.0)	
Sex				
Male	7.5 (7.0-7.9)	<.001	3.0 (2.7-3.3)	<.001
Female	13.8 (13.3-14.4)		7.2 (6.8-7.7)	
Age, y				
18-29	11.3 (10.5-12.2)	.002	2.7 (2.4-3.2)	<.001
30-39	12.7 (11.8-13.7)		5.5 (4.8-6.1)	
40-49	10.0 (9.2-10.9)		5.1 (5.0-5.7)	
50-59	11.9 (11.0-12.8)		6.8 (6.1-7.6)	
≥60	8.8 (8.2-9.4)		5.9 (5.4-6.4)	
Household income, US$				
<25 000	10.6 (9.8-11.5)	.002	4.9 (4.4-5.5)	.57
25 000-49 999	10.9 (10.2-11.6)		5.5 (5.0-6.1)	
50 000-99 999	11.6 (11.0-12.3)		5.6 (5.1-6.1)	
100 000-149 000	10.5 (9.6-11.5)		5.0 (4.3-5.7)	
≥150 000	8.8 (7.7-10.0)		4.0 (3.3-5.7)	
Born in the United States				
Yes	10.8 (10.5-11.2)	.37	5.1 (4.9-5.4)	.06
No	10.2 (8.9-11.6)		5.5 (4.6-6.7)	
Census region				
West	11.5 (10.7-12.3)	.07	5.4 (4.9-6.0)	.43
Midwest	10.3 (9.6-11.0)		4.9 (4.4-5.4)	
South	10.4 (9.9-11.0)		5.0 (4.7-5.5)	
Northeast	11.2 (10.3-12.2)		5.5 (4.8-6.3)	
Physician-diagnosed comorbid conditions				
Asthma	20.9 (19.5-22.3)	<.001	9.9 (9.0-10.9)	.77
Atopic dermatitis or eczema	19.2 (17.4-21.1)	<.001	9.0 (7.8-10.4)	.66
Environmental allergies	17.2 (16.3-18.2)	<.001	10.0 (9.3-10.8)	<.001
Insect sting allergy	22.9 (20.5-25.6)	<.001	13.4 (11.5-15.6)	<.001
Latex allergy	28.8 (25.5-32.3)	<.001	18.4 (15.6-21.5)	<.001
Medication allergy	18.5 (17.3-19.8)	<.001	11.3 (10.4-12.4)	<.001
Urticaria or chronic hives	27.8 (22.9-33.3)	<.001	18.8 (14.6-23.8)	<.001
Other chronic conditions	12.7 (11.4-14.2)	.003	7.5 (6.5-8.7)	<.001

Abbreviations: FA, food allergy; NA, not applicable.

The 5 most common convincing food allergies reported among adults were shellfish (2.9%; 95% CI, 2.7%-3.1%), peanut (1.8%; 95% CI, 1.7%-1.9%), milk (1.9%; 95% CI, 1.8%-2.1%), tree nut (1.2%; 95% CI, 1.1%-1.3%), and fin fish (0.9%; 95% CI, 0.8%-1.0%) (Table 2). Multiple convincing food allergies were reported by 45.3% (95% CI, 43.6%-47.1%) of convincingly food-allergic adults (Table 3). Roughly half of adults with convincing food allergies reported having a physician-diagnosed convincing food allergy (47.5%; 95% CI, 45.8%-49.3%). Individuals with peanut allergy reported the highest rate of physician diagnosis (72.5% [95% CI, 68.9%-75.8%] of convincing peanut allergies).

**Table 2.** Overall and Age-Specific Prevalence of Specific Food Allergies Among All US Adults

Specific Food Allergy	Prevalence, % (95% CI)						
	All Ages	18-29 y	30-39 y	40-49 y	50-59 y	≥60 y
Any food allergy	10.8 (10.4-11.1)	11.3 (10.5-12.2)	12.7 (11.8-13.7)	10.0 (9.2-10.9)	11.9 (11.0-12.8)	8.8 (8.2-9.4)
Peanut	1.8 (1.7-1.9)	2.5 (2.2-2.8)	2.9 (2.5-3.3)	1.8 (1.5-2.1)	1.4 (1.1-1.7)	0.8 (0.7-1.0)
Tree nut	1.2 (1.1-1.3)	1.6 (1.3-1.9)	1.7 (1.4-2.1)	1.1 (0.9-1.4)	1.2 (0.9-1.5)	0.6 (0.4-0.7)
Walnut	0.6 (0.6-0.7)	0.8 (0.7-1.1)	0.9 (0.7-1.3)	0.6 (0.5-0.8)	0.7 (0.5-0.9)	0.3 (0.2-0.4)
Almond	0.7 (0.6-0.8)	0.9 (0.7-1.2)	1.0 (0.7-1.3)	0.7 (0.6-1.0)	0.7 (0.5-0.9)	0.3 (0.2-0.4)
Hazelnut	0.6 (0.5-0.7)	0.7 (0.5-0.9)	0.9 (0.6-1.2)	0.6 (0.4-0.8)	0.6 (0.4-0.8)	0.3 (0.2-0.4)
Pecan	0.5 (0.5-0.6)	0.6 (0.5-0.8)	0.8 (0.5-1.1)	0.6 (0.5-0.8)	0.5 (0.4-0.8)	0.5 (0.4-0.8)
Cashew	0.5 (0.5-0.6)	0.8 (0.6-1.0)	0.8 (0.6-1.1)	0.5 (0.4-0.7)	0.5 (0.3-0.7)	0.2 (0.1-0.3)
Pistachio	0.4 (0.3-0.5)	0.6 (0.4-0.8)	0.6 (0.4-0.8)	0.5 (0.3-0.6)	0.4 (0.3-0.6)	0.1 (0.1-0.2)
Other tree nut	0.2 (0.1-0.2)	0.1 (0.1-0.2)	0.1 (0.0-0.2)	0.3 (0.2-0.6)	0.2 (0.1-0.5)	0.1 (0.1-0.2)
Milk	1.9 (1.8-2.1)	2.4 (2.0-2.9)	2.3 (1.9-2.8)	2.0 (1.6-2.4)	1.9 (1.6-2.2)	1.9 (1.6-2.2)
Shellfish	2.9 (2.7-3.1)	2.8 (2.4-3.2)	3.6 (3.1-4.2)	2.5 (2.2-3.0)	3.3 (2.8-3.8)	2.6 (2.2-3.0)
Shrimp	1.9 (1.8-2.1)	1.8 (1.5-2.1)	2.5 (2.1-3.0)	1.8 (1.4-2.1)	2.2 (1.8-2.6)	1.6 (1.3-1.9)
Lobster	1.3 (1.2-1.4)	1.2 (1.0-1.5)	1.6 (1.3-2.0)	1.3 (1.0-1.5)	1.4 (1.1-1.7)	1.1 (0.9-1.3)
Crab	1.3 (1.2-1.5)	1.2 (1.0-1.5)	1.6 (1.3-2.0)	1.3 (1.0-1.6)	1.6 (1.3-2.0)	1.1 (0.9-1.4)
Mollusk	1.6 (1.4-1.7)	1.6 (1.3-2.0)	2.0 (1.7-2.5)	1.3 (1.1-1.7)	1.7 (1.4-2.0)	1.2 (1.0-1.5)
Other shellfish	0.3 (0.2-0.3)	0.3 (0.1-0.5)	0.1 (0.1-0.2)	0.3 (0.2-0.4)	0.3 (0.2-0.5)	0.3 (0.2-0.4)
Egg	0.8 (0.7-0.9)	1.1 (0.7-1.5)	1.1 (0.9-1.3)	0.7 (0.5-0.9)	0.8 (0.6-1.1)	0.5 (0.3-0.7)
Fin fish	0.9 (0.8-1.0)	1.1 (0.9-1.4)	1.0 (0.8-1.2)	0.8 (0.6-1.1)	1.0 (0.7-1.3)	0.6 (0.4-0.7)
Wheat	0.8 (0.7-0.9)	1.0 (0.7-1.3)	1.0 (0.8-1.3)	0.8 (0.6-1.0)	0.7 (0.5-0.9)	0.6 (0.4-0.8)
Soy	0.6 (0.5-0.7)	0.7 (0.5-0.9)	0.8 (0.6-1.0)	0.6 (0.5-0.8)	0.7 (0.5-0.9)	0.4 (0.3-0.6)
Sesame	0.2 (0.2-0.3)	0.3 (0.2-0.4)	0.3 (0.2-0.5)	0.2 (0.1-0.4)	0.3 (0.2-0.5)	0.1 (0.0-0.2)

**Table 3.** Allergen-Specific FA Characteristics and Health Care Utilization Among Adults With Convincing FA

Specific FA	Prevalence, % (95% CI)^a^						
	Severe Reaction	Adult-Onset FA	Multiple FAs	Physician Diagnosed	Current Epinephrine Prescription	Lifetime History of FA-Related ED Visits	Past 12-mo History of FA-Related ED Visits
All allergens	51.1 (49.3-52.9)	48.0 (46.2-49.7)	45.3 (43.6-47.1)	47.5 (45.8-49.3)	24.0 (22.6-25.4)	38.3 (36.7-40.0)	8.6 (7.7-9.6)
Peanut	67.8 (64.2-71.1)	17.5 (14.8-20.7)	67.8 (64.1-71.3)	72.5 (68.9-75.8)	53.8 (49.9-57.6)	62.3 (58.6-65.9)	19.8 (17.1-22.9)
Tree nut	61.3 (56.6-65.8)	34.6 (30.1-39.4)	90.4 (87.5-92.6)	61.4 (56.6-65.9)	51.5 (46.7-56.2)	54.3 (49.5-59.0)	19.2 (15.6-23.5)
Walnut	51.1 (44.6-57.6)	26.6 (20.8-33.2)	95.1 (92.2-97.0)	53.3 (46.7-59.7)	51.0 (44.5-57.5)	57.0 (50.5-63.4)	18.7 (13.5-25.4)
Almond	57.2 (50.8-63.3)	26.7 (21.4-32.8)	95.7 (92.8-97.5)	63.0 (56.6-69.0)	55.3 (48.7-61.8)	60.7 (54.5-66.7)	24.5 (19.1-30.9)
Hazelnut	55.1 (47.8-62.2)	25.9 (19.8-33.0)	96.2 (92.2-98.2)	58.0 (50.8-64.9)	54.0 (46.6-61.3)	60.6 (53.4-67.3)	19.7 (14.0-26.9)
Pecan	51.4 (44.0-58.6)	29.5 (22.7-37.4)	100	53.2 (45.8-60.4)	56.3 (48.7-63.6)	56.3 (48.9-63.5)	20.1 (14.4-27.3)
Cashew	50.6 (43.6-57.5)	27.7 (21.3-35.2)	96.3 (93.1-98.0)	57.1 (50.2-63.8)	59.3 (52.1-66.1)	58.4 (51.5-65.0)	21.4 (15.7-28.4)
Pistachio	49.6 (41.5-57.7)	28.1 (21.7-35.6)	97.0 (93.9-98.6)	57.9 (49.9-65.5)	56.8 (48.2-65.0)	63.4 (55.7-70.5)	20.9 (14.3-29.6)
Other tree nut	59.7 (44.6-73.1)	30.9 (19.0-46.1)	80.8 (65.7-90.3)	43.0 (29.1-58.1)	52.7 (37.8-67.1)	43.9 (29.7-59.1)	4.5 (1.6-11.7)
Milk	39.3 (35.2-43.5)	22.7 (19.6-26.3)	60.1 (55.9-64.2)	47.1 (43.0-51.3)	24.0 (20.9-27.5)	47.0 (42.8-51.1)	12.0 (9.9-14.4)
Shellfish	56.8 (53.4-60.1)	48.2 (44.8-51.6)	69.9 (66.5-73.2)	42.1 (39.0-45.4)	27.4 (24.7-30.3)	45.3 (42.0-48.7)	11.1 (9.0-13.5)
Shrimp	56.6 (52.6-60.5)	37.2 (33.3-41.3)	76.1 (72.1-79.7)	42.6 (38.8-46.5)	29.8 (26.5-33.4)	47.7 (43.8-51.7)	10.6 (8.6-13.0)
Lobster	48.3 (43.5-53.1)	40.5 (35.8-45.5)	94.1 (91.3-96.1)	35.9 (31.5-40.5)	32.8 (28.6-37.4)	53.0 (48.2-57.8)	12.5 (9.6-16.1)
Crab	48.9 (44.2-53.5)	40.0 (35.4-44.7)	89.7 (86.1-92.4)	35.1 (30.9-39.5)	32.8 (28.7-37.2)	51.9 (47.2-56.6)	11.3 (8.6-14.7)
Mollusk	47.0 (42.4-51.6)	39.2 (34.7-43.8)	81.0 (76.5-84.8)	33.1 (29.2-37.2)	30.3 (26.4-34.5)	50.8 (46.2-55.4)	12.4 (9.3-16.4)
Other shellfish	60.1 (49.6-69.7)	39.2 (29.3-50.0)	89.8 (80.2-95.1)	28.8 (19.9-39.7)	35.9 (25.9-47.4)	50.9 (40.0-61.6)	10.7 (4.6-22.7)
Egg	39.4 (32.8-46.5)	29.0 (23.2-35.6)	65.6 (58.3-72.1)	52.1 (45.1-59.0)	34.0 (28.5-40.0)	55.0 (47.8-61.9)	22.4 (17.6-28.0)
Fin fish	56.5 (51.0-61.7)	39.9 (34.7-45.4)	89.8 (86.2-92.5)	40.9 (35.7-46.3)	37.2 (32.1-42.6)	60.1 (54.7-65.3)	19.9 (15.9-24.7)
Wheat	42.6 (36.2-49.3)	52.6 (46.1-59.0)	68.3 (61.8-74.1)	55.5 (48.9-61.9)	24.6 (20.0-29.9)	43.6 (37.3-50.1)	14.9 (11.1-19.8)
Soy	45.4 (38.9-52.2)	45.4 (38.8-52.2)	81.2 (75.4-85.9)	48.5 (41.9-55.2)	37.3 (31.4-43.6)	48.3 (41.7-55.1)	18.2 (13.6-23.9)
Sesame	39.7 (30.3-49.9)	25.7 (18.1-35.1)	80.3 (67.5-88.9)	37.7 (28.7-47.6)	61.6 (51.3-70.9)	66.2 (54.6-76.2)	31.5 (23.1-41.5)

Abbreviations: ED, emergency department; FA, food allergy.

^a^ All columns represent frequency with a denominator of all those with convincing FA to each specified food.

### Food Allergy Severity and Health Care Use 

Among adults with 1 or more convincing food allergies, 51.1% (95% CI, 49.3%-52.9%) reported experiencing at least 1 severe food-allergic reaction (Table 3). A history of severe reactions was most commonly observed among participants with convincing peanut (67.8%; 95% CI, 64.2%-71.1%) and tree nut (61.3%; 95% CI, 56.6%-65.8%) allergies. Among adults with 1 or more convincing food allergies, 24.0% (95% CI, 22.6%-25.4%) reported a current epinephrine prescription and 38.3% (95% CI, 36.7%-40.0%) reported 1 or more lifetime food allergy–related ED visits. A total of 8.6% (95% CI, 7.7%-9.6%) of convincingly food-allergic adults reported 1 or more food allergy–related ED visit within the past year.

### Factors Associated With Food Allergies and Related Conditions

**Figure 2.**
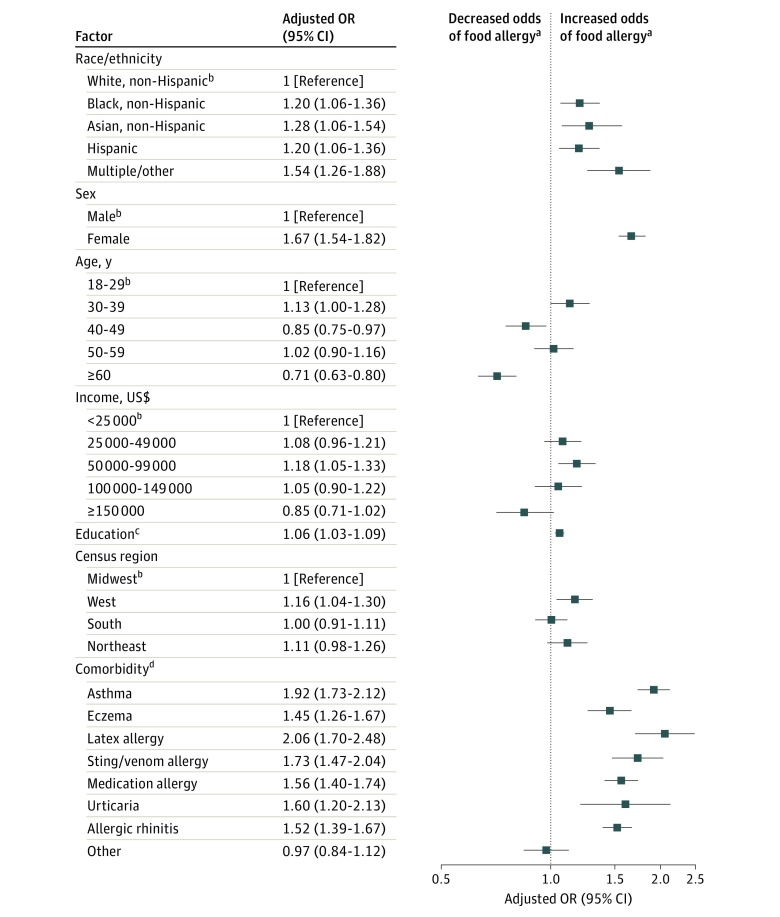
Factors Associated With Current Food Allergy Each square represents the odds ratio (OR) point estimate for each corresponding variable or sample characteristic, adjusting for all other variables in the logistic regression model. Each horizontal line represents the 95% CI. Percentages of all adults in each subgroup and adults with current food allergies in each subgroup are given in eTable 1 in the Supplement. ^a^Compared with the reference group. ^b^Reference group. ^c^Educational attainment was modeled as a continuous variable with the following 7 categories: less than high school, high school, some college, associates, bachelors, masters, and professional or doctorate. ^d^The reference group for each comorbid condition is the absence of that condition.

Adjusted associations from multiple logistic regression models estimating odds of convincing food allergy and food allergy characteristics are presented in eTable 2 in the Supplement. Significant differences in convincing food allergy prevalence were observed by race/ethnicity, with higher rates among groups other than white compared with white adults. Rates of convincing food allergy were higher among females (13.8%; 95% CI, 13.3%-14.4%) compared with males (7.5%; 95% CI, 7.0%-7.9%). Compared with younger adults, individuals aged 30 to 39 years had elevated rates of convincing food allergy (12.7%; 95% CI, 11.8%-13.7%), whereas rates were lower for those 60 years or older (8.8%; 95% CI, 8.2%-9.4%). In adjusted models, each assessed chronic atopic comorbidity, including asthma, eczema, allergic rhinitis, urticaria, and latex allergy, was significantly associated with increased odds of convincing food allergy (Figure 2).

Adults were more likely to have a physician-diagnosed convincing food allergy if they earned $25 000 or more annually compared with those earning less than $25 000. Having multiple convincing food allergies, a current epinephrine prescription, a history of 1 or more lifetime food allergy–related ED visits, a severe reaction history, comorbid allergic rhinitis, or latex allergies were each associated with increased odds of having 1 or more physician-diagnosed convincing food allergy. When examining factors related to a severe food allergy reaction history, convincingly food-allergic adults older than 50 years had significantly decreased risk of severe food allergy compared with younger adults, whereas black adults (odds ratio [OR], 1.4; 95% CI, 1.1-1.7) and adults with comorbid asthma (OR, 1.4; 95% CI,1.1-1.6) or allergic rhinitis (OR, 1.3; 95% CI, 1.1-1.5) were at increased risk for severe food allergy.

### Factors Associated With Epinephrine Prescription and ED Visits

eTable 3 in the Supplement reports factors associated with having a current epinephrine prescription, reporting 1 or more lifetime food allergy–related ED visits, and reporting 1 or more food allergy–related ED visits within the past year. Adults reporting 1 or more lifetime ED visits (OR, 3.2; 95% CI, 2.6-3.9) or severe food allergy (OR, 1.5; 95% CI, 1.2-1.8) had elevated odds of having a current epinephrine prescription, as did adults with peanut (OR, 2.4; 95% CI, 1.9-3.1), tree nut (OR, 3.3; 95% CI, 2.0-5.3), sesame (OR, 3.0; 95% CI, 1.4-6.2), or soy allergy (OR, 1.5; 95% CI, 1.0-2.1) or a comorbid insect sting allergy (OR, 2.0; 95% CI, 1.4-2.9). Adults 50 years or older also had significantly reduced odds of a current epinephrine prescription. Current epinephrine prescription rates varied considerably by food allergy type, with the highest rates observed among adults with sesame (61.6%), peanut (53.8%), or tree nut allergy (51.5%). With respect to lifetime ED visits, adults with multiple food allergies (OR, 1.2; 95% CI, 1.0-1.5), severe food allergy (OR, 1.9; 95% CI, 1.6-2.3), childhood-onset food allergy only (OR, 1.7; 95% CI, 1.4-2.0), a current epinephrine prescription (OR, 3.2; 95% CI, 2.6-3.9), or comorbid asthma (OR, 1.3; 95% CI, 1.0-1.5) had significantly elevated odds of 1 or more food allergy–related ED visits, as did Hispanics and adults earning less than $25 000 per year.

## Discussion

The present population-weighted data revealed that an estimated 10.8% of US adults had at least 1 current food allergy during the study period (corresponding to >26 million US adults), whereas 19.0% of adults believed that they were food allergic. These data suggest that there are currently at least 13 million food-allergic adults who have experienced at least 1 severe food-allergic reaction, at least 10 million adults who have received food allergy treatment in the ED, and at least 12 million adults with adult-onset food allergy.

This overall estimate of adult food allergy prevalence falls between the 10% estimated from 2007-2010 National Health and Nutrition Examination Survey data by McGowan and Keet^9^ and estimates reported by Verrill et al^10^ from 2010 FDA Food Safety Survey data, who reported an overall adult food allergy prevalence of 13% and physician-diagnosed food allergy prevalence of 6.5%. However, neither of these previous surveys collected data on reaction symptoms that could be used to identify adults reporting food allergies that are unlikely to be IgE mediated. Given that the most prevalent allergies observed were shellfish and peanut, which prior pediatric work suggests are infrequently outgrown,^25^ this finding suggests that the population-level burden of food allergy is likely to increase in the future, absent widespread implementation of effective prevention efforts and/or therapies. Of interest, the current data suggest that shellfish allergy may be a particularly enduring allergy among adults. For example, estimated shellfish allergy prevalence was 2.8% among individuals aged 18 to 29 years and 2.6% among those 60 years or older, a lower rate of decrease across the life span than observed for other food allergies. These relatively high rates of shellfish allergy across the life span, including adult-onset shellfish allergies, require further investigation. Whether these high rates are attributable to different underlying pathophysiological mechanisms among shellfish-allergic patients, greater awareness of shellfish allergy, and/or additional factors remains to be seen and is the subject of ongoing research. Shellfish has long been acknowledged as a persistent allergy,^8,26,27^ although adult cohort studies are needed to more definitively establish its natural history.

Among US adults, our data revealed that the burden of shellfish allergy was greatest, affecting an estimated 7.2 million US adults. Milk (affecting an estimated 4.7 million adults), peanut (4.5 million), tree nut (3.0 million), fin fish (2.2 million), egg (2.0 million), wheat (2.0 million), soy (1.5 million), and sesame (0.5 million) were the next most common food allergies.

As summarized in a recent review,^28^ racial/ethnic disparities in allergic diseases, such as asthma^29^ and eczema,^30^ are well established, and data suggest that the burden of child food allergy may also be greater among the population of races/ethnicities other than white, non-Hispanic.^17^ However, much less is known about such disparities in adult food allergy. The current data showed that food allergy rates were significantly higher among adults other than white, even after adjustment for income, educational level, numerous physician-diagnosed atopic conditions, and other covariates. These findings are consistent with findings from our previous population-based study^8,17^ of child food allergy prevalence, which also found elevated rates of food allergy in non-Hispanic black and Asian children. Although previous examinations of food allergy disparities have largely contrasted sensitization and estimated prevalence rates between non-Hispanic black and white populations,^31,32^ the present findings suggest that the scope of future work examining food allergy disparities should be expanded to further investigate racial/ethnic differences among Hispanic adults. In the current study, Hispanic adults were estimated to have comparable rates of food allergy to non-Hispanic black adults, as well as the highest rates of food allergy–related ED visits among all racial groups, despite reporting epinephrine prescription rates comparable to those of white adults.

Clinical food allergy management guidelines recommend intramuscular epinephrine as first-line treatment for food-induced anaphylaxis.^33^ All patients diagnosed with a food allergy should be prescribed epinephrine because of the inability to accurately and reliably estimate the severity of future allergic reactions.^34,35^ Our data suggest that approximately one-quarter of adults with food allergy possess a current epinephrine prescription, with higher rates among adults reporting a history of severe reactions and lifetime food allergy–related ED visits. These overall rates of epinephrine prescription are comparable to the 23% of peanut- and tree nut–allergic adults reporting an epinephrine prescription in a 2002 prevalence study.^36^ However, further analyses suggest that a substantial proportion of adults with food allergy who may be at elevated risk of anaphylaxis do not report having a current epinephrine prescription. For instance, among adults with 1 or more severe, physician-diagnosed food allergies who reported at least 1 food allergy–related ED visit in the past year, only 65% reported a current epinephrine prescription. These low rates of epinephrine possession are particularly notable given that nearly 40% of food-allergic adults reported at least 1 lifetime food allergy–related ED visit and more than half reported a history of 1 or more severe food-allergic reactions.

The high rate of severe reactions in our study compared with previous literature^17^ is consistent with findings from multiple studies^37,38,39^ showing an association of increased age with more severe allergic reaction symptoms. However, it is also possible that the higher proportion of adults reporting severe reactions is a function of adults’ greater cumulative lifetime risk. This idea is supported by the slightly reduced rates of severe reactions and ED visits observed among adults reporting adult-onset food allergy in the present study. More specifically, the significantly elevated odds of severe food allergy observed among adults with comorbid allergic rhinitis extends findings from a large case series where a marked increase in food-induced severe pharyngeal edema was observed among peanut- and tree nut–allergic patients with comorbid allergic rhinitis.^40^ Although less than 10% of food-allergic adults reported a food allergy–related ED visit within the past year, this figure increased to 32% among sesame-allergic adults, who also reported the highest epinephrine possession rates in the cohort (62% vs 24% overall). Patients with comorbid asthma were also at increased risk of food allergy–related ED visits, which is consistent with previous work that found an association of asthma with increased anaphylaxis risk.^41^

Adult-onset food allergies are an important emerging health problem. A recent analysis^13^ of electronic health record data collected from a network of Chicago-area clinics concluded that although shellfish, tree nut, and fin fish allergies were the most common adult-onset food allergies, it appears to be possible to develop adult-onset food allergies to all major food allergen groups. In the current study, adult-onset allergies were observed to every assessed food. After wheat, the most common adult-onset allergies in our sample were shellfish, soy, tree nut, and fin fish, which were the top 4 allergies identified by Kamdar et al.^13^ Furthermore, the observed rates of adult-onset shellfish and fin fish allergy in our sample are not dissimilar to the rates of 60% and 40%, respectively, observed by Sicherer et al^8^ more than a decade ago. The most common childhood-onset allergy was peanut, which underlines the importance of early-life primary prevention efforts, such as the targeted early introduction practices advocated by the recent Addendum Guidelines for the Prevention of Peanut Allergy in the United States.^42^

In light of the considerable economic^1^ and quality of life^3^ consequences associated with allergen avoidance and other food allergy management behaviors, individuals with a suspected food allergy should receive appropriate confirmatory testing and counseling to counter unnecessary avoidance of allergenic food. Greater patient education efforts regarding key differences between food intolerances and allergies also may be warranted.^43^ Furthermore, the results of our study suggest that adults need to be encouraged to see their physicians to receive proper diagnosis, epinephrine prescription, and counseling for their food allergy. Given the increasing evidence for the preventive benefits of early allergen exposure during infancy and potential treatment options, adults should be made aware of these new practices to potentially prevent food allergies in their children or consider treatments in the near future.

### Limitations

Although double-blinded, placebo-controlled oral food challenges remain the criterion standard for food allergy diagnosis, such methods were not used to confirm self-reported food allergy in the present study because of their expense and impracticality with such a large nationally representative sample and concerns about nonparticipation bias. However, similar to past work,^7^ to strengthen the rigor of our self-report questionnaire, stringent criteria were established in collaboration with an expert panel to exclude food allergies for which corresponding symptom report was not consistent with an IgE-mediated food allergy. Nevertheless, given the self-report paradigm used in the present study, bias remains a concern.

## Conclusions

These data suggest that at least 1 in 10 US adults are food allergic. However, they also suggest that nearly 1 in 5 adults believe themselves to be food allergic, whereas only 1 in 20 are estimated to have a physician-diagnosed food allergy. Overall, approximately half of all food-allergic adults developed at least 1 adult-onset allergy, suggesting that adult-onset allergy is common in the United States among adults of all ages, to a wide variety of allergens, and among adults with and without additional, childhood-onset allergies.

## Notes

Supplement. **eMethods.** Dual-Sample Complex Survey Sampling and Weighting Methodology **eFigure.** List of Allergic Reaction Symptoms Highlighting Stringent Symptoms Indicative of Convincing Food Allergy **eTable 1.** Demographic Distribution of Sample, Food-Allergic Adults, Adult- and Childhood-Onset Allergies [Frequency % (95% CI)] **eTable 2.** Demographic and Allergic Characteristics Associated With Convincing, Physician-Diagnosed, Adult-Onset, Severe, and Multiple Food Allergies, Adjusted Odds Ratio (95% CI) **eTable 3.** Demographic and Allergic Characteristics Associated With Epinephrine Prescription, Lifetime ED Visits, Last Year ED Visits, Adjusted Odds Ratio (95% CI)
