# Supplementary material for: Prevalence and Severity of Food Allergies Among US Adults
Source: JAMA Netw Open. 2019 Jan 4;2(1):e185630. doi: 10.1001/jamanetworkopen.2018.5630 (PMC6324316; doi:10.1001/jamanetworkopen.2018.5630)
Supplement: Supplement. — eMethods. Dual-Sample Complex Survey Sampling and Weighting Methodology eFigure. List of Allergic Reaction Symptoms Highlighting Stringent Symptoms Indicative of Convincing Food Allergy eTable 1. Demographic Distribution of Sample, Food-Allergic Adults, Adult- and Childhood-Onset Allergies [Frequency % (95% CI)] eTable 2. Demographic and Allergic Characteristics Associated With Convincing, Physician-Diagnosed, Adult-Onset, Severe, and Multiple Food Allergies, Adjusted Odds Ratio (95% CI) eTable 3. Demographic and Allergic Characteristics Associated With Epinephrine Prescription, Lifetime ED Visits, Last Year ED Visits, Adjusted Odds Ratio (95% CI) [file jamanetwopen-2-e185630-s001.pdf]

Supplementary Online Content

Gupta RS, Warren CM, Smith BM, et al. Prevalence and severity of food allergies among US adults. *JAMA Netw Open.* 2019;2(1): e185630. doi:10.1001/jamanetworkopen.2018.5630

eMethods. Dual-Sample Complex Survey Sampling and Weighting Methodology

eFigure. List of Allergic Reaction Symptoms Highlighting Stringent Symptoms Indicative of Convincing Food Allergy

eTable 1. Demographic Distribution of Sample, Food-Allergic Adults, Adult- and Childhood-Onset Allergies [Frequency % (95% CI)]

eTable 2. Demographic and Allergic Characteristics Associated With Convincing, Physician-Diagnosed, Adult-Onset, Severe, and Multiple Food Allergies, Adjusted Odds Ratio (95% CI)

eTable 3. Demographic and Allergic Characteristics Associated With Epinephrine Prescription, Lifetime ED Visits, Last Year ED Visits, Adjusted Odds Ratio (95% CI)

This supplementary material has been provided by the authors to give readers additional information about their work.

© 2019 Gupta RS et al. *JAMA Network Open.*

eMethods. Dual-sample Complex Survey Sampling and Weighting Methodology

Overview of Survey Sampling and Weighting Methods Utilized

Participant recruitment and survey administration were conducted by NORC at the University of Chicago—an independent national survey research organization founded in 1941 as the National Opinion Research Center. As in our 2011 national pediatric food allergy prevalence study, a dual-sample approach was employed. This approach systematically combines survey data obtained from probability- and non-probability-based samples via a best-in-class statistical technique for calibrating and adjusting survey estimates to offset error introduced though the non-probability sample. Briefly, a bivariate Fay-Herriot small area model was used to model food allergy prevalence rates from the probability-based *AmeriSpeak* sample and the non-probability-based *SSI* samples. By incorporating an additive bias term for each domain, this model was used to estimate the bias in the overall food allergy prevalence rate associated with the SSI sample, and then to adjust the SSI sample weights to the "bias corrected" overall food allergy prevalence estimate. The reliability and validity of this methodological approach to combine probability and non-probability samples using "small area estimation" is well-documented[1,2,3]. Consequently, small-area estimation is often used by the US Census Bureau, Centers for Disease Control, Bureau of Labor Statistics, and other leading survey research organizations to provide reliable statistics for small areas or subpopulations where sample sizes are too small to obtain sufficiently accurate estimates [4].

[1] Ganesh, N., Pineau, V., Chakraborty, A., Dennis, JM. Probability and Non-Probability Samples Using Small Area Estimation. JSM Proceedings 201

[2] Elliott, M.N. and Haviland, A. 2007. Use of a web-based convenience sample to supplement a probability sample. Survey Methodology 33(2): 211-215.

[3] Jiang, J. and Lahiri, P. Mixed model prediction and small area estimation (with discussion). Test 15(1):1–96. 2006.

[4] Rao, J.N.K. and Molina, I. 2015. Small area estimation. Wiley, 2nd Edition

© 2019 Gupta RS et al. *JAMA Network Open.*

NORC's Probability-based *AmeriSpeak Panel*

To obtain our target sample size of 40,000 adults, first a target of 7,210 participants was initially recruited from NORC's web-enabled AmeriSpeak® panel. This target sample size was selected to be consistent with a prior childhood food allergy prevalence study[5] where power analyses determined that such a sample size would provide .90 power (assuming a significance level of .05) to detect: (1) overall and allergen-specific food allergy prevalence rates from 1% to 9%; and (2) variability in prevalence estimates from 1% to 7% among groups as small as 1% of the overall analytic sample. In the present study, a survey completion rate of 51.2% was observed among AmeriSpeak panelists (7,210 completions among 14,095 sampled units). The weighted cumulative AAPOR response rate for the *AmeriSpeak* sample was 8.8%. This rate is a function of the 18.3% rate of sampled households successfully recruited into the *AmeriSpeak* panel, the 93.8% rate of successfully recruited households who were also successfully retained into the panel, and the aforementioned 51.2% completion rate among successfully recruited, and retained *AmeriSpeak* Panelists who were approached for this survey. The AmeriSpeak panel is a socio-demographically and geographically-representative sample of US households recruited via a probability-based sample design using NORC's National Frame. This allows for derivation of unbiased estimates representing the full population of interest with known margins of error accounting for the sample design, response rates, and survey weighting.

[5] Gupta RS, Springston EE, Warrier MR, Smith B, Kumar R, Pongracic J, Holl JL. The prevalence, severity, and distribution of childhood food allergy in the United States. Pediatrics. 2011 Jul;128(1):e9-17
.

© 2019 Gupta RS et al. *JAMA Network Open.*

AmeriSpeak Panel Sampling Frame

In order to provide a nationally representative sample, *AmeriSpeak* leverages the NORC National Sample Frame, constructed by NORC to cover over 97 percent of U.S. households. The 2010 National Frame used a two-stage probability sample design to select a representative sample of households in the United States. The first stage—the sampling unit—is a National Frame Area (NFA), which is either an entire metropolitan area (made up of one or more counties) or a county (some counties were combined so that each NFA contains a population of at least 10,000). The largest NFAs with a population of at least 1,543,728 (0.5 percent of the 2010 Census U.S. population) were selected with certainty; these areas have a high-population density, and are dominated by tracts with street-style addresses. These areas contain 56 percent of the population within 8 percent of the geographic area of the United States. The remaining areas were stratified into areas where street-style addresses predominate, and the remaining areas, which are less likely to have street -style addresses. The latter stratum ("rural" areas) comprises 81 percent of the geographic area, but only 14 percent of the population. Within the selected NFAs, the second stage sampling unit is a segment, defined either in terms of Census tracts or block groups, containing at least 300 housing units according to the 2010 Census. A stratified probability sample of 1,514 segments was selected with probability proportional to size. For most of the 1,514 segments, the USPS DSF provided over 90 percent coverage of the segment in terms of city-style addresses that are geo-codeable. For the 123 segments where the DSF provided insufficient coverage, we enhanced the DSF address list with in-person listing. The National Sample Frame contains almost 3 million households, including over 80,000 rural households added through the in-person listing. AmeriSpeak incorporated address-based sampling (ABS) addresses in 2016 from the USPS DSF to assure AmeriSpeak sample representation within all US States. As of October 2016, 0.9% of AmeriSpeak Panel recruited adults were sourced from the ABS (99.1% from the National Frame). Proper weights, such as those employed in the present study, allow the full use of the combined sample.

© 2019 Gupta RS et al. *JAMA Network Open.*

Sample Selection for AmeriSpeak Panel Recruitment

As summarized above, the 2014-2016 *AmeriSpeak* Panel sample consists of nationally representative housing units drawn from the 2010 NORC National Sample Frame and less than 1% from address-based sampling. The 2010 NORC National Sample Frame is stratified based on segment (Census tract or Census block group) characteristics such as age and race/ethnicity composition of the segment, and then, a stratified simple random sample of housing units is selected. Specifically, based on Census tract-level data, segments were classified as having a higher concentration of 18-24 year old adults or not, and a higher concentration of Hispanics, non-Hispanic African Americans, and other. Based on these strata definitions, 6 strata (2 based on age times 3 based on race/ethnicity) were used to oversample housing units in segments higher in young adults and/or Hispanics and non-Hispanic African-Americans. This is referred to as the initial sample or first stage of panel recruitment.

In the second stage of panel recruitment, initially sampled but nonresponding housing units are subsampled for a nonresponse follow-up (NRFU). At this stage, consumer vendor data are matched to housing units, and housing units that are flagged (based on consumer vendor data) as having a young adult or minority (Hispanic and non-Hispanic African American) are oversampled for the nonresponse follow-up. Overall, approximately one in five initially nonresponding housing units are subsampled for NRFU. However, as mentioned previously, selection of housing units for NRFU is a stratified simple random sample based on consumer vendor data. Due to NRFU, these initially nonresponding housing units have a much higher selection probability compared to the housing units that were recruited during the first stage of panel recruitment. Note that a small fraction of initially nonresponding housing units are not eligible for NRFU due to these housing units being classified as “hard refusals” or having an appointment for a call back from NORC.

© 2019 Gupta RS et al. *JAMA Network Open.*

In summary, there are two reasons why the sampling design for *AmeriSpeak* Panel recruitment deviates from equal probability of selection method sampling: (a) oversampling of housing units in segments with a higher concentration of young adults and minorities results in the sample selection probabilities being higher for housing units in these segments; and (b) the nonresponse follow-up effort results in initially nonresponding housing units having a much higher selection probability. Furthermore, oversampling associated with NRFU results in higher selection probabilities for initially nonresponding housing units that are flagged (based on consumer vendor data) as having a young adult or minority.

AmeriSpeak Panel Recruitment Procedures.

Recruitment is a two-stage process: initial recruitment using less expensive methods and then non-response follow-up using personal interviewers. For the initial recruitment, sample units are invited to join *AmeriSpeak* online by visiting the panel website AmeriSpeak.org or by telephone (in-bound/outbound supported). English and Spanish language are supported for both online and telephone recruitment. Study invitations are communicated via an over-sized pre-notification postcard, a USPS recruitment package in a 9”x12” envelope (containing a cover letter, a summary of the privacy policy, FAQs, and a study brochure), two follow-up post cards, and also follow-up by NORC’s telephone research center for matched sample units.

The second-stage non-response follow-up targets a stratified random sub-sample of the non-responders from the initial recruitment. Stratification is based on consumer vendor data and stratification variables from the initial recruitment stage in order to increase sample representation of young adults, non-Hispanic African Americans, and Hispanics. Units sampled for the non-response follow-up are sent by Federal Express a new recruitment package with an enhanced incentive offer. NORC field interviewers then make personal, face-to- face visits to the respondents’ homes to encourage

© 2019 Gupta RS et al. *JAMA Network Open.*

participation. NORC field interviewers administer the recruitment survey in-person using CAPI or else encourage the respondents to register at AmeriSpeak.org or call the toll-free *AmeriSpeak* telephone number to register.

Impact of Non-Response Follow-up on *AmeriSpeak* Panel Representativeness

The non-response follow-up improves the representativeness of the AmeriSpeak sample with respect to certain demographic segments, including but not limited to rural and/or lower income households, cell- phone only households, persons age 18 to 34, African Americans, Hispanics, and persons without a high school degree or only have a high school degree (no college). Compared to panelists recruited in the initial stage, panelists recruited via the non-response follow-up campaign are more politically conservative, are less knowledgeable about science, report less interest in current events and topics in the news (such as climate change), and are less likely to read a print newspaper.

AmeriSpeak Panel Implementation of Mixed-Mode Data Collection

Panelists may participate in 2 to 3 *AmeriSpeak* Panel studies per month via online (computer, tablet, or smartphones) or by CATI phone. NORC maintains strict rules to limit respondent burden and reduce the risk of panel fatigue. CATI phone mode respondents represent a population currently under- represented in web panels that exclude non-internet households or “net averse” persons. NORC’s telephone interviewers administer the phone mode of survey questionnaires using a data collection system supporting both the CATI phone and web modes of data collection, providing an integrated sample management and data collection platform. For panelists using smartphones for web-mode *AmeriSpeak* surveys,

© 2019 Gupta RS et al. *JAMA Network Open.*

the NORC survey system renders an optimized presentation of the survey questions for these mobile users. For general population client studies, approximately 20% of the completed interviews are completed by the telephone mode.

Survey Sampling International's Non-Probability-based Sample

To augment the probability-based sample recruited from NORC's AmeriSpeak Panel, participants were also recruited from an online non-probability-based sample of US households administered by Survey Sampling International (SSI). A response rate of 5.5% of invited adults was observed. Due to the opt-in nature of the non-probability- based SSI sample, and corresponding lack of a national sampling frame an AAPOR response rate cannot be calculated. Non-probability opt-in samples do not generally allow for derivation of unbiased estimates due to the lack of randomness in the opt-in sample and the high likelihood that the opt-in panel is skewed toward a non-representative subset of individuals, thus leading to an increased likelihood of biased estimates from the opt-in sample. Nevertheless, SSI employs a variety of approaches to limit bias, including use of invitations of all types to recruit participants with a diversity of motivations. These include e-mail invitations, telephone alerts to mobile devices, banners and messaging on SSI panel community sites. The messages themselves are also varied, including invitations to give your opinion, win a prize, earn cash or prizes or let your voice be heard. A diversity of motivation contributes to high-quality sample. Furthermore, to avoid self-selection bias, specific study details were not included in the invitation. Rather, participants were invited to "take a survey". The details are disclosed later, when the participant initiates the survey.

Complex Survey Weighting Details

Responses from the *AmeriSpeak*® panel were used to identify and correct for sampling and non-response biases via calculation and application of probability sampling weights. Following the nonresponse adjustment, iterative proportional

© 2019 Gupta RS et al. *JAMA Network Open.*

fitting (also known as “raking”) was conducted in order to rake survey weights to national US population totals associated with age, sex, education, race/ethnicity, and Census Division.

A base weight of one was assigned to each SSI non-probability sample complete, and then iterative proportional fitting was used to rake the non-probability sample weights to national US population totals associated with age, sex, education, race/ethnicity, “early adopter” characteristics, Census Division, and the bias-corrected overall food allergy prevalence estimates calculated using the nationally-representative *AmeriSpeak* sample. Finally, the probability and non-probability samples are combined through derivation of an optimal composition factor that minimizes mean square error associated with FA prevalence estimates.

Comparing Distributions of Unweighted *AmeriSpeak* respondents, Weighted *AmeriSpeak* respondents, and US Census Bureau Demographics

The below table demonstrates the high degree to which the 7210 *AmeriSpeak* respondents in the present study are demographically representative of the general US population. Specifically, the figure compares the demographic distributions of *AmeriSpeak* respondents before and after our weighting approach was implemented, and then compares the weighted distributions to national demographics reported in the February 2016 Current Population Survey.

Comparing Unweighted and Weighted *AmeriSpeak* Respondent Demographics with 2016 US Census Bureau Current Population Survey Estimates

© 2019 Gupta RS et al. *JAMA Network Open.*

	Unweighted	Weighted	Benchmark	Difference
Household Income				1.6
Less than $30,000	28.1	21.7	21.7	0
$30,000 to $74,000	37.9	33.8	34.7	0.9
$75,000 to $124,999	22	26	22.8	3.2
$125,000 Plus	12	18.4	20.7	2.3
Member Age				0
18 - 34	22.7	30.2	30.2	0
35 - 49	23.3	25	25	0
50 - 64	31.6	25.9	25.9	0
65 Plus	22.5	19	19	0
Race/Ethnicity				1.1
White	67	64.9	64.9	0
Black	15	11.7	11.7	0
Hispanic	9.6	15.5	15.5	0
Asian/Pacific Islander	2.8	3.2	6	2.8
Others	5.7	4.8	2	2.8
Education Status				4.4
Less than High School	4.8	6.7	12.2	5.5
High School Equivalent	16.3	24.1	29.6	5.5
Some College	37.2	30.2	28.4	1.8
Bachelor's Degree	23.8	22	19.2	2.8
Graduate Degree	17.9	17	10.6	6.4
Household Ownership				2.4
Owner Occupied	64.4	69.4	67	2.4
Renter Occupied/Other	35.6	30.6	33	2.4
Children in Household				0.2
With 1+ Under 18 Years	30	35.6	35.8	0.2
Without Children Under 18	70	64.4	64.2	0.2
Marital Status				0.9
Currently Married	48.7	52	52.9	0.9
Currently Single	51.3	48	47.1	0.9
Sex				0
Male	42.8	48.3	48.3	0
Female	57.2	51.7	51.7	0
Average Difference				1.3

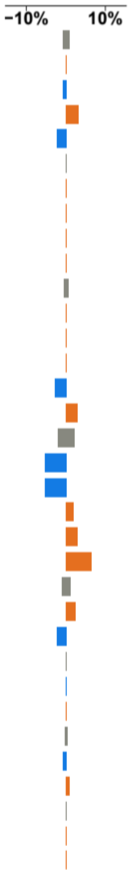

© 2019 Gupta RS et al. *JAMA Network Open.*

eFigure. List of allergic reaction symptoms highlighting stringent symptoms indicative of convincing food allergy

- □ Skin/Oral Mucosa Symptoms
  - □ ***Hives***
  - □ Itching
  - □ Rash
  - □ ***Swelling*** (except lip/tongue swelling)
  - □ ***Lip/tongue swelling***
  - □ ***Difficulty swallowing***
  - □ Hoarse voice
  - □ Itchy mouth
  - □ ***Throat tightening***
  - □ Mouth or throat tingling
  - □ Other: ___________________
- □ Respiratory Symptoms
  - □ ***Chest tightening***
  - □ Nasal congestion
  - □ Repetitive cough
  - □ ***Trouble breathing***
  - □ ***Wheezing***
  - □ Other: ___________________
- □ Gastrointestinal (GI) Symptoms
  - □ Belly pain
  - □ Cramps
  - □ Diarrhea
  - □ Nausea
  - □ ***Vomiting***
  - □ Other: ___________________
- □ Cardiovascular/Heart Symptoms
  - □ ***Chest pain***
  - □ ***Rapid heart rate***
  - □ ***Fainting, dizziness, or feeling light headed***
  - □ ***Low blood pressure***
  - □ Other: __________________
- □ Other Symptoms
  - □ Anxiety
  - □ Feeling of impending doom
  - □ Headache
  - □ Other: ___________________

All symptoms listed are offered as answer choices in the survey. Symptoms in bold italics comprised our expert panel's stringent symptom list.
A "convincing" food allergy required the report of at least one stringent symptom during the participant's most severe reaction to a given food.

© 2019 Gupta RS et al. *JAMA Network Open.*

eTable 1. Demographic Distribution of Sample, Food-allergic Adults, Adult- and Childhood-onset Allergies [Frequency % (95% CI)]

Variable	All Adults	Adults with Current FA	p-value	Adults with ≥1 Adult-onset FA	Adults with Childhood-onset FA only	p-value
Race/ethnicity						
Asian, non-Hispanic	3.9 (3.6-4.1)	4.1 (3.5-4.8)	<.001	3.6 (2.8-4.5)	4.6 (3.7-5.6)	<.001
Black, non-Hispanic	11.7 (11.3-12.1)	12.2 (11.1-13.3)		11.5 (10.0-13.2)	12.7 (11.2-14.4)	
White, non-Hispanic	64.9 (64.2-65.6)	61.1 (59.3-62.9)		65.4 (62.9-67.9)	57.1 (54.6-59.5)	
Hispanic	15.5 (14.9-16.1)	16.6 (15.2-18.2)		13.8 (11.8-16.0)	19.3 (17.2-21.4)	
Multiple/other	4.1 (3.8-4.4)	6.0 (5.1-7.1)		5.7 (4.6-7.1)	6.4 (5.0-8.0)	
Sex						
Female	51.7 (51.0-52.4)	66.4 (64.8-68.1)	<0.001	72.3 (69.9-74.5)	61.1 (58.7-63.4)	<0.001
Male	48.3 (47.6-49.0)	33.6 (31.9-35.2)		27.7 (25.5-30.1)	38.9 (36.6-41.3)	
Age						
18-29 Years	21.5 (20.8-22.1)	22.6 (21.1-24.1)	<0.001	11.4 (9.9-13.0)	32.9 (30.6-35.2)	<0.001
30-39 Years	17.0 (16.5-17.5)	20.1 (18.7-21.5)		18.9 (16.0-20.0)	22.0 (20.1-24.1)	
40-49 Years	16.8 (16.3-17.3)	15.6 (14.4-16.9)		16.5 (14.7-18.5)	14.8 (13.3-16.5)	
50-59 Years	18.0 (17.5-18.5])	19.9 (18.5-21.4)		23.8 (21.6-26.1)	16.3 (14.6-18.2)	
60+ Years	26.8 (26.2-27.4)	21.9 (20.4-23.3)		30.5 (28.1-32.9)	13.9 (12.4-15.6)	
Household income, $						
<25,000	16.6 (16.2-17.1)	16.3 (15.2-17.6)	0.002	15.8 (14.3-17.5)	16.8 (15.1-18.7)	0.57
25,000-49,999	22.0 (21.4-22.5)	22.2 (20.9-23.6)		23.4 (21.5-25.5)	21.1 (19.4-22.9)	
50,000-99,999	30.9 (30.3-31.5)	33.4 (31.8-35.0)		33.4 (31.0-35.9)	33.3 (31.2-35.5)	
100,000-149,000	19.6 (19.0-20.2)	19.1 (17.6-20.8)		18.8 (16.7-21.2)	19.4 (17.3-21.7)	
>150,000	10.9 (10.4-11.5)	8.9 (7.9-10.1)		8.5 (7.1-10.3)	9.3 (7.8-11.0)	
Born in the US						
Yes	91.6 (91.2-92.0)	92.0 (90.9-93.0)	0.37	91.0 (89.2-92.5)	93.0 (91.5-94.3)	0.06
No	8.4 (8.1-8.8)	8.0 (7.0-9.1)		9.0 (7.5-10.8)	7.0 (5.7-8.5)	
Geographic location						
West	23.4 (22.9-24.2)	25.1 (23.6-26.7)	.07	24.5 (22.4-26.8)	23.5 (22.9-24.1)	.43
Midwest	20.9 (20.4-21.4)	20.0 (18.7-21.3)		19.8 (18.0-21.8)	21.0 (20.5-21.5)	
South	37.4 (36.7-38.1)	36.1 (34.5-37.8)		36.5 (34.1-39.0)	37.5 (36.8-38.2)	
Northeast	18.1 (17.6-18.7)	18.8 (17.4-20.4)		19.2 (17.0-21.6)	18.1 (17.5-18.6)	

© 2019 Gupta RS et al. *JAMA Network Open.*

Variable	All Adults	Adults with Current FA	p-value	Adults with ≥1 Adult-onset FA	Adults with Childhood-onset FA only	p-value
Physician Diagnosed Comorbid Conditions						
Asthma	12.3 (11.8-12.7)	23.8 (22.3-25.3)	<0.001	23.5 (21.5-25.7)	24.0 (22.0-26.2)	0.77
Atopic Dermatitis/Eczema	6.7 (6.4-7.1)	12.0 (10.9-13.2)	<0.001	11.7 (10.2-13.5)	12.2(10.7-14.0)	0.66
Environmental Allergies	21.4 (20.9-22.0)	34.3 (32.7-36.0)	<0.001	41.5 (39.0-44.1)	27.6 (25.6-29.8)	<0.001
Insect Sting Allergy	3.8 (3.6-4.1)	8.1 (7.2-9.1)	<0.001	9.9 (8.5-11.5)	6.5 (5.4-7.7)	<0.001
Latex Allergy	2.3 (2.1-2.5)	6.1 (5.4-7.0)	<0.001	8.2 (6.9-9.7)	4.3 (3.5-5.2)	<0.001
Medication Allergy	13.4 (13.0-13.9)	23.1 (21.6-24.6)	<0.001	29.4 (27.2-31.7)	17.2 (15.5-19.1)	<0.001
Urticaria/Chronic Hives	0.9 (0.8-1.0)	2.2 (1.8-2.8)	<0.001	3.2 (2.4-4.1)	1.4 (1.0-1.9)	<0.001
Other Chronic Conditions	7.3 (7.0-7.7)	8.6 (7.7-9.6)	0.003	10.6 (9.2-12.2)	6.8 (5.7-8.1)	<0.001

© 2019 Gupta RS et al. *JAMA Network Open.*

eTable 2. Demographic and allergic characteristics associated with convincing, physician-diagnosed, adult-onset, severe, and multiple food allergies, Adjusted Odds Ratio (95% CI)

Variable	**Convincing FA** vs. No FA	**Physician-diagnosed FA** vs. Convincing FA	**One or more adult-onset FA** vs. childhood-onset only	**One or more severe FA** vs. Mild-to-Moderate only	**Multiple FA** vs. Single FA
Race/ethnicity (vs White, non-Hispanic)					
Asian, non-Hispanic	1.3 (1.1-1.5)	1.0 (0.7-1.4)	1.0 (0.7-1.4)	1.0 (0.7-1.5)	0.6 (0.4-0.9)
Black, non-Hispanic	1.2 (1.1-1.4)	1.0 (0.8-1.3)	0.9 (0.7-1.1)	1.4 (1.1-1.7)	1.2 (0.9-1.5)
Hispanic	1.2 (1.1-1.4)	0.9 (0.7-1.2)	0.9 (0.7-1.2)	1.0 (0.8-1.3)	1.1 (0.9-1.4)
Multiple/other	1.5 (1.3-1.9)	0.7 (0.5-1.0)	1.0 (0.7-1.4)	1.2 (0.8-1.8)	0.7 (0.5-1.0)
***Sex* (vs. Male)**					
Female	1.7 (1.5-1.8)	0.9 (0.7-1.0)	1.7 (1.4-2.0)	0.9 (0.7-1.0)	1.0 (0.8-1.1)
Age (vs. 18-29 years)					
30-39	1.1 (1.0-1.3)	0.9 (0.7-1.2)	2.5 (2.0-3.2)	0.8 (0.6-1.0)	0.9 (0.7-1.1)
40-49	0.9 (0.8-1.0)	0.8 (0.6-1.0)	3.5 (2.7-4.5)	0.9 (0.7-1.2)	0.8 (0.6-1.0)
50-59	1.0 (0.9-1.2)	0.7 (0.5-0.9)	4.3 (3.3-5.5)	0.7 (0.6-0.9)	0.7 (0.6-0.9)
60+	0.7 (0.6-0.8)	0.6 (0.5-0.8)	5.7 (4.4-7.3)	0.7 (0.5-0.8)	0.7 (0.5-0.9)
Annual Household income, $					
25,000-49,999	1.1 (1.0-1.2)	1.4 (1.1-1.8)	1.1 (0.9-1.4)	1.0 (0.8-1.2)	1.1 (0.9-1.4)
50,000-99,999	1.2 (1.0-1.3)	1.8 (1.4-2.3)	1.0 (0.8-1.2)	0.9 (0.7-1.1)	1.1 (0.9-1.4)
100,000-149,000	1.0 (0.9-1.2)	1.4 (1.1-2.0)	0.8 (0.6-1.1)	1.1 (0.8-1.5)	1.0 (0.7-1.3)
>150,000	0.9 (0.7-1.0)	1.5 (1.1-2.2)	0.7 (0.5-0.9)	1.0 (0.7-1.4)	1.0 (0.7-14)
Educational Attainment [a]	1.1 (1.0-1.1)	1.0 (1.0-1.1)	1.1 (1.1-1.2)	0.9 (0.8-0.9)	1.1 (1.0-1.1)
Geographic location (vs Midwest)					
West	1.2 (1.0-1.3)	1.0 (0.8-1.3)	1.2 (0.9-1.5)	0.8 (0.7-1.0)	0.9 (0.7-1.2)
South	1.0 (0.9-1.1)	1.1 (0.9-1.3)	1.2 (1.0-1.5)	0.9 (0.7-1.0)	0.8 (0.7-1.0)
Northeast	1.1 (1.0-1.3)	1.0 (0.8-1.3)	1.2 (1.0-1.6)	0.8 (0.7-1.1)	1.0 (0.7-1.2)

© 2019 Gupta RS et al. *JAMA Network Open.*

Variable	**Convincing FA** vs. No FA	**Physician-diagnosed FA** vs. Convincing FA	**One or more adult-onset FA** vs. childhood-onset only	**One or more severe FA** vs. Mild-to-Moderate only	**Multiple FA** vs. Single FA
Comorbid Conditions (vs. absence of that condition)					
Asthma	1.9 (1.7-2.1)	1.0 (0.8-1.2)	0.9 (0.8-1.1)	1.4 (1.1-1.6)	1.1 (0.9-1.4)
Atopic Dermatitis/Eczema	1.5 (1.3-1.7)	1.2 (1.0-1.6)	0.9 (0.7-1.1)	0.8 (0.6-1.0)	1.2 (1.0-1.6)
Allergic Rhinitis	1.5 (1.3-1.7)	1.2 (1.0-1.5)	1.3 (1.1-1.5)	1.3 (1.1-1.5)	1.1 (0.9-1.4)
Insect Sting Allergy	1.7 (1.5-2.0)	1.0 (0.7-1.3)	0.9 (0.7-1.2)	1.1 (0.8-1.4)	1.4 (1.1-1.9)
Latex Allergy	2.1 (1.7-2.5)	1.5 (1.1-2.1)	1.4 (1.1-1.9)	1.1 (0.8-1.5)	1.5 (1.1-2.0)
Medication Allergy	1.6 (1.4-1.7)	0.9 (0.8-1.2)	1.3 (1.1-1.6)	0.8 (0.7-1.0)	1.0 (0.8-1.2)
Urticaria/Chronic Hives	1.6 (1.2-2.1)	1.5 (0.9-2.6)	1.7 (1.0-2.8)	0.9 (0.6-1.5)	0.8 (0.5-1.3)
Other Chronic Condition	1.0 (0.8-1.1)	1.0 (0.7-1.3)	1.0 (0.7-1.3)	1.4 (1.0-1.8)	1.2 (0.9-1.5)
**Physician FA Diagnosis** ***(vs. no physician-diagnosed FA)***	N/A	N/A	0.8 (0.7-0.9)	1.3 (1.1-1.5)	2.1 (1.7-2.5)
**Multiple FA** ***(vs. single FA)***	N/A	2.1 (1.7-2.4)	2.0 (1.7-2.3)	2.8 (2.4-3.3)	N/A
**Current Epinephrine Prescription** ***(vs. not)***	N/A	3.4 (2.8-4.2)	0.8 (0.7-1.0)	1.7 (1.4-2.0)	1.4 (1.2-1.7)
**1 or more Lifetime ED Visit** ***(vs. 0 lifetime ED visits)***	N/A	3.0 (2.5-3.5)	0.6 (0.5-0.7)	2.0 (1.7-2.3)	1.5 (1.3-1.8)
**1 or more Severe FA** ***(vs. 0 severe FA)***	N/A	1.3 (1.1-1.5)	1.0 (0.9-1.2)	N/A	2.8 (2.4-3.3)
**1 or more adult-onset FA** ***(vs. childhood-onset FA only)***	N/A	0.8 (0.7-0.9)	N/A	1.0 (0.9-1.2)	2.0 (1.7-2.3)

[a] Response categories for educational attainment were as follows: Less Than High School, High School, Some College, Associates, Bachelors, Masters, Professional/Doctorate

© 2019 Gupta RS et al. *JAMA Network Open.*

eTable 3: Demographic and allergic characteristics associated with Epinephrine Prescription, Lifetime ED Visits, Last Year ED Visits, Adjusted Odds Ratio (95% CI)

Variable	**Current Epinephrine Prescription** vs. No Current Epinephrine Prescription	**Lifetime ED Visits** vs. No Lifetime ED Visits	**Last Year ED Visits** vs. No Last Year ED Visits
Race/ethnicity (vs White, non-Hispanic)			
Asian, non-Hispanic	0.7 (0.4-1.3)	1.1 (0.7-1.6)	0.9 (0.6-1.5)
Black, non-Hispanic	0.8 (0.6-1.1)	1.3 (1.0-1.7)	1.2 (0.8-1.8)
Hispanic	0.9 (0.7-1.2)	1.5 (1.2-1.9)	1.3 (0.9-1.9)
Multiple/other	0.8 (0.6-1.2)	1.0 (0.7-1.4)	0.8 (0.4-1.4)
**Sex *(vs. Male)***			
Female	1.0 (0.8-1.2)	1.1 (0.9-1.3)	0.9 (0.7-1.1)
**Age *(vs. 18-29 years)***			
30-39 Years	0.8 (0.6-1.0)	1.4 (1.1-1.7)	1.1 (0.8-1.5)
40-49 Years	0.8 (0.6-1.0)	1.7 (1.3-2.3)	0.6 (0.4-0.9)
50-59 Years	0.6 (0.4-0.8)	1.7 (1.3-2.3)	0.5 (0.3-0.8)
60+ Years	0.5 (0.3-0.6)	1.3 (1.0-1.8)	0.5 (0.3-1.0)
**Household income *(vs <$25K/year)***			
25K-49K	1.2 (0.9-1.6)	0.7 (0.5-0.9)	0.5 (0.3-0.8)
50K-99K	1.5 (1.1-2.0)	0.7 (0.6-0.9)	0.5 (0.3-0.7)
100K-149K	1.8 (1.3-2.6)	0.5 (0.4-0.7)	0.3 (0.2-0.5)
150K+	1.5 (0.9-2.4)	0.6 (0.4-0.8)	0.5 (0.3-0.8)
**Educational Attainment**	1.1 (1.1-1.2)	1.0 (0.9-1.0)	0.9 (0.8-1.0)
**Geographic location *(vs Midwest)***			
West	0.9 (0.7-1.1)	1.1 (0.8-1.4)	1.1 (0.7-1.6)
South	1.0 (0.8-1.3)	1.3 (1.0-1.6)	1.0 (0.7-1.5)
Northeast	1.2 (0.9-1.7)	0.9 (0.7-1.2)	1.0 (0.7-1.6)
**Comorbid Conditions *(vs. absence of that condition)***			
Asthma	1.2 (1.0-1.5)	1.3 (1.0-1.5)	1.4 (1.1-1.9)
Atopic Dermatitis/Eczema	1.0 (0.8-1.4)	0.7 (0.6-1.0)	0.9 (0.5-1.5)
Environmental Allergies	1.0 (0.8-1.2)	0.8 (0.7-1.0)	0.7 (0.5-1.1)
Insect Sting Allergy	2.0 (1.4-2.9)	1.3 (0.9-1.7)	1.0 (0.6-1.7)
Latex Allergy	1.1 (0.8-1.6)	1.1 (0.8-1.5)	0.7 (0.4-1.3)
Medication Allergy	1.1 (0.8-1.4)	1.2 (1.0-1.5)	0.5 (0.4-0.8)
Urticaria/Chronic Hives	1.4 (0.8-2.3)	1.4 (0.9-2.4)	1.9 (0.9-3.8)
Other Chronic Condition	0.6 (0.4-0.8)	0.9 (0.7-1.2)	1.1 (0.7-1.8)
**Physician FA Diagnosis *(vs. no physician-diagnosed FA)***	3.2 (2.6-4.0)	2.8 (2.4-3.4)	1.8 (1.3-2.5)

© 2019 Gupta RS et al. *JAMA Network Open.*

**Multiple FA** ***(vs. single FA)***	1.0 (0.8-1.3)	1.2 (1.0-1.5)	1.2 (0.9-1.7)
**Variable**	**Current Epinephrine Prescription** vs. No Current Epinephrine Prescription	**Lifetime ED Visits** vs. No Lifetime ED Visits	**Last Year ED Visits** vs. No Last Year ED Visits
**Current Epinephrine Prescription** ***(vs. no current epinephrine prescription)***	N/A	3.2 (2.6-3.9)	2.4 (1.8-3.2)
**1 or more Lifetime ED Visit** ***(vs. 0 lifetime ED visits)***	3.2 (2.6-3.9)	N/A	N/A
**1 or more Severe FA** ***(vs. 0 severe FA)***	1.5 (1.2-1.8)	1.9 (1.6-2.3)	1.2 (0.9-1.7)
**1 or more adult-onset FA** ***(vs. childhood-onset FA only)***	0.9 (0.7-1.1)	0.6 (0.5-0.7)	1.3 (1.0-1.8)
**Specific Food Allergies** ***(vs. absence of that allergy)***			
Peanut	2.4 (1.9-3.1)	1.5 (1.2-1.9)	1.7 (1.3-2.2)
Tree Nut	3.3 (2.0-5.3)	0.8 (0.5-1.2)	1.3 (0.7-2.4)
Almond	0.8 (0.5-1.3)	1.3 (0.8-2.0)	1.9 (1.0-3.4)
Walnut	0.5 (0.3-0.9)	1.1 (0.7-1.7)	0.9 (0.5-1.6)
Cashew	1.3 (0.8-2.1)	0.8 (0.5-1.2)	1.0 (0.5-1.8)
Hazelnut	0.8 (0.5-1.3)	1.3 (0.8-2.1)	0.8 (0.4-1.4)
Pistachio	0.9 (0.5-1.5)	1.3 (0.8-2.1)	0.7 (0.4-1.4)
Pecan	1.2 (0.7-2.2)	0.8 (0.5-1.3)	1.0 (0.6-1.8)
Other Tree Nut	2.0 (0.8-5.3)	0.6 (0.3-1.3)	0.4 (0.1-1.2)
Sesame	3.0 (1.4-6.2)	1.5 (0.9-2.7)	2.1 (1.2-3.8)
Milk	0.8 (0.6-1.5)	1.4 (1.1-1.8)	1.3 (1.0-1.8)
Egg	1.1 (0.8-1.5)	1.4 (1.0-2.0)	2.3 (1.5-3.3)
Finfish	1.1 (0.8-1.6)	1.6 (1.2-2.1)	1.9 (1.2-2.8)
Shellfish	0.9 (0.6-1.4)	1.1 (0.7-1.6)	1.8 (1.0-3.2)
Shrimp	1.2 (0.8-1.8)	1.0 (0.7-1.4)	0.7 (0.4-1.1)
Lobster	0.9 (0.6-1.5)	1.0 (0.7-1.6)	1.1 (0.7-1.8)
Crab	1.3 (0.8-2.0)	1.1 (0.8-1.6)	0.7 (0.4-1.2)
Mollusk	1.1 (0.7-1.7)	1.3 (0.9-1.9)	1.1 (0.7-1.8)
Other Shellfish	1.4 (0.7-2.9)	0.7 (0.4-1.4)	0.9 (0.4-2.2)
Soy	1.5 (1.0-2.1)	0.8 (0.5-1.1)	1.1 (0.7-1.7)
Wheat	0.6 (0.4-0.9)	1.0 (0.7-1.4)	1.5 (0.9-2.3)

© 2019 Gupta RS et al. *JAMA Network Open.*
